# Analysis of macerated ticks using Boolean logic gating colorimetric isothermal nucleic acid assays for Lyme *Borrelia* and *Ixodes scapularis* ticks

**DOI:** 10.1038/s41598-023-38452-8

**Published:** 2023-07-15

**Authors:** Sanchita Bhadra, Maria D. Esteve-Gasent, Andrew D. Ellington

**Affiliations:** 1 Department of Molecular Biosciences, College of Natural Sciences, The University of Texas at Austin, Austin, TX USA; 2 Center for Systems and Synthetic Biology, The University of Texas at Austin, Austin, TX USA; 3 Department of Veterinary Pathobiology, College of Veterinary Medicine and Biomedical Sciences, Texas A&M University, College Station, TX USA

**Keywords:** Analytical biochemistry, Infectious-disease diagnostics

## Abstract

Lyme disease, one of the most common tickborne diseases, has been rapidly spreading in parallel with the expansion of the range of its tick vector. Better tick surveillance efforts are needed to accurately estimate disease risk and to guide public health and clinical management. We have developed two multiplex loop-mediated isothermal amplification (LAMP) reactions coupled with oligonucleotide strand displacement (OSD) probes to identify the tick host, *Ixodes scapularis*, and the Lyme disease pathogen, *Borrelia burgdorferi*, they carry. In each multiplex LAMP-OSD assay the co-presence of two target sequences is computed at the DNA level by linking the two corresponding amplicons and detecting the co-product on colorimetric lateral flow dipsticks. In tests with synthetic DNA, the co-presence of as few as four copies of input DNA could be detected, without producing spurious signals. Most importantly, though, the LAMP-OSD assay is amenable to being carried out directly with macerated tick samples, without any sample preparation. In such field conditions, assays performed robustly and demonstrated 97–100% sensitivity and 100% specificity with both field-collected and lab-raised artificially infected ticks. Such easy-to-use, arthropod and pathogen-specific assays would be well suited to field and near patient use without relying on complex instrumentation or infrastructure.

## Introduction

Lyme disease (LD) is the most common tick-borne disease in the Northern hemisphere with an annual incidence of over 476,000 cases in the United States (US) alone^1^. It is primarily caused by *Borrelia* species, obligate parasitic spirochete bacteria that are maintained by enzootic transmission between *Ixodes* ticks and vertebrates, including mammals, reptiles, and birds^2,3^. Three genospecies in the *Borrelia burgdorferi* sensu lato (sl) bacterial group—*B. garinii*, *B. afzelii*, and *B. burgdorferi* sensu stricto (ss)—are responsible for most cases. While all three genospecies are prevalent in Europe, in the US LD is principally caused by *B. burgdorferi* ss^1,3^. Recently a new genospecies, *B. mayonii*, was found to be associated with LD cases in the upper Midwestern US^4,5^. Another *B. burgdorferi* sl genospecies, *B. bissettii*, found mostly in the Western and Southeastern parts of US, might also instigate LD^6^. The *Ixodid* tick hosts are naturally infected with *B. burgdorferi* and are often co-infected by more than one genospecies.

Early detection of infection can lead to antibiotic treatments that are typically curative. However, if left undiagnosed LD frequently develops into disseminated disease that can affect neurologic, cardiac, and musculoskeletal systems. Unfortunately, early diagnosis is often confounded by non-specific symptoms and lack of risk awareness^7^. Since increased local density of infected ticks correlates well with human disease incidence, *B. burgdorferi* surveillance can help inform comprehensive spatiotemporal models of vector and pathogen distribution that estimate disease endemicity and risk of spread^8,9^. These data in turn can be used to raise LD awareness, heighten personal protection and community-wide vector control programs, and aid accurate diagnosis of LD by engendering aggressive monitoring of early clinical signs and molecular markers of LD. Unfortunately, current US-wide tick surveillance efforts are sorely lacking^8^. Improving such efforts is a challenge, since morphological identification of ticks requires expertise and is often confounded by similar appearance, such as between *I. scapularis* and *I. affinis*, and sympatric distribution, which makes distinction difficult, especially in the nymph stage^10^. Such distinctions are critical, especially since human transmissions in eastern and north-central US are primarily caused by the ‘human-biting’ *I. scapularis*, while *I. pacificus* is the main disseminator along the West coast^11^. Due to factors such as globalization, climate warming, and changing predator populations, the geographic range of *I. scapularis* has been rapidly expanding^12–17^. Concomitantly, the number and distribution of LD cases has been steadily increasing over the past few decades, with LD being increasingly reported in historically non-endemic areas^18,19^. For instance, *I. scapularis* and *B. burgdorferi* have been detected in Canada^20^ and LD ‘hotspots’ have emerged with 380% increased incidence in Minnesota, 280% rise in Wisconsin, and 1300% jump in Virginia^21^.

While molecular methods can readily distinguish different tick and bacterial species^10,20,22–25^, current molecular techniques, such as those based on immunoassays or the polymerase chain reaction (PCR), are complex, and require technical expertise and extensive infrastructure for accuracy. Sloppy execution of such tests and the resulting false positives have led to unnecessary treatment and even death^26,27^. In consequence, samples are typically shipped to centralized molecular testing laboratories, which slows down turnaround time and hampers accessibility.

The wide expanse and environmental vagaries of tick distribution and the fact that most human encounters occur in less urbanized areas drives a need for innovative, dependable, and cost-effective molecular diagnostic tools. Herein we report a simple and rapid molecular diagnostic for direct testing of crudely crushed ticks detecting genetic signatures identifying tick species and *B. burgdorferi* sl. This molecular diagnostic is comprised of two nucleic acid tests, one specific for *Ixodes scapularis* and the other for *B. burgdorferi*. Both tests use multiplex LAMP to co-amplify two target sequences that are coupled into a single colorimetric readout via sophisticated DNA computation^28^. Despite the complex underlying molecular machinery, users can simply use crushed ticks as an input for the assay and then read the presence of molecular markers directly via colorimetric ‘yes/no’ answers on a lateral flow dipstick.

In trials with field-collected and lab-infected ticks, the *I. scapularis* assay demonstrated 97% sensitivity in species identification, while the *B. burgdorferi* assay had 100% sensitivity in identifying bacterial pathogens. Both assays demonstrated 100% diagnostic specificity. The accuracy and simplicity of the assay potentiates use on-site by otherwise untrained individuals. This molecular diagnostic should prove useful not only for the widespread surveillance of LD but may be readily reconfigured for testing other infectious agents in ticks known to transmit the largest variety of bacterial and viral pathogens, being ultimately responsible for spreading ~ 95% of vector-borne diseases reported in the US^29^.

## Materials and methods

### Chemicals and reagents

All reagents were of analytical grade and obtained from Sigma-Aldrich (St. Louis, MO, USA) unless otherwise indicated. Bst 2.0 DNA polymerase and isothermal amplification buffer were acquired from New England Biolabs (NEB, Ipswich, MA, USA). All oligonucleotides were obtained from Integrated DNA Technologies (IDT, Coralville, IA, USA). OSD probe strands were subjected to HPLC or polyacrylamide gel purification.

### LAMP-OSD assay design

#### LAMP primer design

*Ixodes scapularis* and *B. burgdorferi* gene sequences were accessed from National Center for Biotechnology Information (NCBI) GenBank and sequence alignments and comparisons within species and to related species were performed using Multiple Sequence Comparison by Log-Expectation (MUSCLE)^30^ and the Basic Local Alignment Search Tool (BLAST)^31,32^. Conserved regions in *I. scapularis* mitochondrial 16S rRNA gene (m16S) and the internal transcribed spacer 2 (ITS) of the nuclear 5.8S rRNA gene and in *B. burgdorferi* 16S rRNA gene (b16S) and the Flagellin gene (Fla) were chosen as targets for amplification by LAMP. The web-based Primer Explorer v5 software (Eiken Chemical Co, Toghigi, Japan) was used to generate LAMP primer sets for the four target sequences (Table S1). Primer specificity for the chosen targets and a corresponding lack of significant cross-reactivity to other nucleic acids was assessed using BLAST.

#### OSD probe design

##### OSD probes for fluorogenic readout

Four hemiduplex OSD probes specific to m16S, ITS, b16S, and Fla LAMP amplicons, respectively, were engineered using our previously published OSD design rules^33^ and the nucleic acid circuit design software NUPACK^34^ (Table S1). The fluorescein (FAM) labeled OSD long strands were complementary to the target-specific sequence between B1c and B2c regions of *I. scapularis* ITS and m16S amplicons and between F1c and F2c sequences of *B. burgdorferi* b16S and Fla amplicons. The Iowa Black FQ (IABkFQ) quencher labeled OSD short strands were complementary to the fluorophore-labeled strand leaving 10 or 12 nucleotides long single-stranded toeholds at the 3’-end or 5’-end of fluorophore-labeled strands (Table S1). Three to four basepair clamps unrelated to the target amplicon were appended at the ends of some OSD duplexes to improve stability. All 3′–OH ends were blocked with inverted deoxythymidine (invdT) to prevent extension by Bst DNA polymerase. Polymorphic loci in the Fla OSD probe were substituted with appropriate degenerate bases. Hemiduplex OSD probes were assembled by combining 1 µM of the long FAM strand with 2 µM of the short quencher strand in 1X isothermal buffer (NEB: 20 mM Tris–HCl, 10 mM (NH4)_2_SO_4_, 50 mM KCl, 2 mM MgSO_4_, 0.1% Tween 20, pH 8.8) and incubating the mixture at 95 °C for 1 min followed by slow cooling at 0.1 °C/sec to 25 °C.

##### Boolean AND-gating strand displacement probes for lateral flow readout

**Figure 1:**
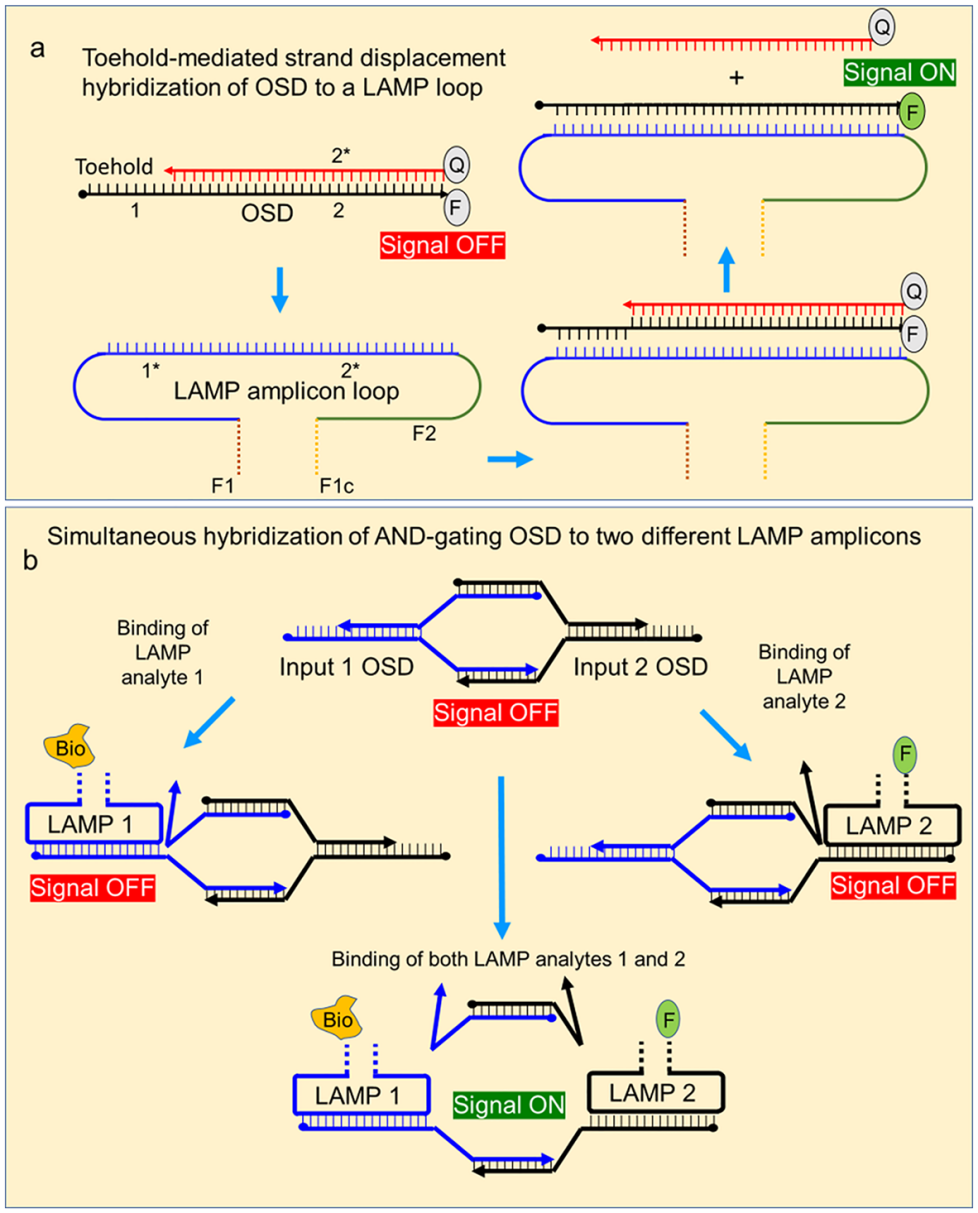
Schematics depicting hybridization of OSD reporters to LAMP amplicons. (**a**) Schematic depicting toehold-mediated strand displacement hybridization of a hemiduplex OSD probe to a single stranded LAMP amplicon loop sequence. Numbers 1 and 2 are domains arbitrarily assigned in the otherwise continuous DNA sequence to represent complementary sequences (indicated by asterisk). Fluorescein is denoted by F while the quencher is denoted as Q. Regions in the LAMP amplicon corresponding to LAMP primer-specific sites are denoted by F1, F1c, and F2. Quenching of signal in the hemiduplex state of the OSD is indicated as a red highlighted ‘signal OFF’ sign. The appearance of fluorescence upon separation of the quencher strand is indicated by the green highlighted ‘signal ON’ sign. (**b**) Schematic depicting detection of amplicon co-presence via AND-gating OSD probes. AND gating OSD designed to bind two different LAMP amplicons remains in the OFF state unless both LAMP amplicons are bound to the probe at the same time. The biotin label on LAMP amplicon 1 is indicated as ‘Bio’ while the fluorescein label on LAMP amplicon 2 is indicated as F.

Two Boolean AND gating strand displacement probes (Table S1) were designed for reporting co-presence of either *I. scapularis* ITS and m16S LAMP amplicons (Ixo-OSD) or *B. burgdorferi* b16S and Fla amplicons (Bor-OSD) on fluorescein-specific lateral flow dipsticks. Both these LFA OSDs were comprised of two long strands, termed L1 and L2, and two short strands, termed S1 and S2, that were designed to be partially complementary to each other (Fig. 1). The Ixo-OSD L1 and L2 strands were derived by replacing the 3’-end fluorescein labels on the m16S and ITS OSD long strands with 52 nucleotide (nt) long random sequences whose terminal 28 bases were complementary to each other. Meanwhile, the Ixo-OSD S1 and S2 strands were generated by substituting the quencher moieties in the m16S and ITS OSD short strands with 53 nt long random sequences whose terminal 28 bases were designed to hybridize with each other. The Bor-OSD was engineered by making similar modifications to the Fla OSD and the reverse complement of the b16S OSD. All free 3′–OH ends were blocked with inverted dT to prevent extension by DNA polymerase. The Ixo- and Bor-OSD complexes were assembled by combining 1 µM each of the corresponding L1, L2, S1, and S2 strands in 1X isothermal buffer and incubating the mixture at 95 °C for 1 min followed by slow cooling at 0.1 °C/sec to 25 °C.

### LAMP-OSD and Mx-LAMP-OSD assays

All LAMP reactions were assembled in 25 µl volume containing 1X isothermal buffer supplemented with 1 M betaine, 0.48 mM deoxyribonucleotide mix, 2 mM additional MgSO_4_, and 16 units (unless otherwise indicated) of Bst 2.0 DNA polymerase. LAMP reactions for amplifying individual targets were supplemented with 200 nM of the long OSD strand annealed with a twofold excess of the short strand and a combination of five primers—1.6 μM each of BIP and FIP primers, 0.8 µM of the loop primer, and 0.4 μM each of B3 and F3 primers. These individual assays were seeded with indicated amounts of synthetic double stranded DNA. For real-time observation of amplification kinetics, these LAMP-OSD assays were incubated in a LightCycler96 real-time PCR machine (Roche, Switzerland) programmed to hold the reactions at 65 °C and measure OSD fluorescence at intervals of 3 min. Endpoint OSD fluorescence was imaged using a ChemiDoc imager (Bio-Rad, Hercules, CA, USA).

AND-gating multiplex LAMP-OSD reactions for simultaneous amplification and lateral flow colorimetric readout of *B. burgdorferi* Fla and b16S sequences contained 200 nM of Bor-OSD, 0.8 µM each of Fla FIP and BIP primers, 0.4 µM each of b16S FIP and BIP primers, 0.4 µM of fluorescein labeled Fla loop primer, 0.2 µM of biotin labeled b16S loop primer, 0.2 µM each of Fla F3 and B3 primers, and 0.1 µM of b16S F3 and B3 primers. Similarly, AND-gating colorimetric multiplex assays for *I. scapularis* m16S and ITS sequences were supplied with 200 nM of Ixo-OSD, 1.6 µM each of m16S FIP and BIP primers, 0.4 µM each of ITS FIP and BIP primers, 0.8 µM of biotinylated m16S loop primer, 0.2 µM of fluorescein labeled ITS loop primer, 0.4 µM each of m16S F3 and B3 primers, and 0.1 µM of ITS F3 and B3 primers. Multiplex assays were seeded with indicated amounts of either synthetic double stranded DNA, purified genomic DNA or macerated tick specimens. Assays were incubated in a 65 °C heat block for 1.5–2 h and then subjected to 1 min incubation at 95 °C. The multiplex reactions were then mixed with 25 µl of lateral flow assay buffer (Milenia Biotec GmbH, Gießen, Germany) followed by room temperature colorimetric readout of AND-gated LAMP amplicons using HybriDetect lateral flow dipsticks (Milenia Biotec) and the manufacturer’s instructions. Images of lateral flow assay dipsticks were acquired using an iPhone 11 cellphone camera with default photo settings and illumination from regular white ceiling lights in the laboratory.

### Preparation of tick specimens for Mx-LAMP-OSD assays

Individual inactivated ticks were received in 1.5 ml microtubes filled with 80% ethanol. Excess ethanol was removed using a pipet and the remaining ethanol was dried by placing the tubes with ticks in a SpeedVac (ThermoFisher Scientific, Waltham, MA, USA) set at medium drying intensity for 30 min. Individual ticks were then transferred to Kimble® BioMasher II® microtubes (DWK Life Sciences, Millville, NJ, USA), hydrated with 20–100 µL of water and manually macerated using the BioMasher II microtube pestle. The macerated ticks were heated at 95 °C for 10 min followed by addition of 2–4 µL of the macerated material directly into Mx-LAMP-OSD reactions.

In some experiments, macerated tick specimens were transferred into Mx-LAMP-OSD reactions using in-house fabricated disposable samplers made of a small thin strip of 3mil self-adhesive plastic (Fellowes, Itasca, IL, USA) at one end of which a 3 mm glass fiber conjugate paper disc (Catalog number GFCP000800, Sigma-Aldrich, St. Louis, MO, USA) was stuck. The remaining plastic strip was folded on itself to form a non-sticky handle using which the paper disc was dipped and swirled in the macerated tick specimen for 1 min. Then the disc was immersed into a 25 µl Mx-LAMP-OSD reaction and the reaction tube was capped such that the disc remained submerged in the reaction throughout amplification while half of the plastic strip handle protruded out of the tube.

### Field collection, lab rearing, and artificial infection of ticks

Preparation of spirochaete cultures and artificial infection of ticks was performed as described previously^35^. Genomic DNA was purified from bacterial cultures following conventional phenol chloroform extractions. Ticks were collected from vegetation in the Big Thicket National Preserve in Texas using conventional dragging technology^36^ and also recovered from animals in Columbus, TX and at the Brazos Valley Humane Society. The collected ticks were stored intact in ethanol at − 20 °C until use.

## Results

### Development of high-surety LAMP-OSD assays for *B. burgdorferi* and *I. scapularis*

In order to identify *I. scapularis* ticks and *B. burgdorferi* bacteria, and thereby improve diagnostic outcomes for LD, we sought to create more specific, portable diagnostic tests for near patient and field use. Since multiplex assays that can simultaneously detect more than one sequence from an organism often improve test accuracy^37,38^, we focused on isothermal amplification assays that relied upon the simultaneous presence of two, separate target-derived amplicons. These amplicons would in turn be transduced into a simple colorimetric readout via a two-input Boolean AND logic gating probe and disposable lateral flow dipsticks, a strategy we have previously used for the successful detection of SARS-CoV-2^39^.

Four LAMP assays with oligonucleotide strand displacement (OSD) probes were implemented: the *B. burgdorferi* Flagellin (Fla) and 16S rRNA genes (b16S), and *I. scapularis* mitochondrial 16S rRNA (m16S) and nuclear 5.8S rRNA internal transcribed spacer 2 (ITS) genes, were each targeted with specific sets of 5 primers. Synthetic, double-stranded DNA targets (400 copies/reaction) were used to evaluate amplification kinetics in real-time via OSD fluorescence, and all four LAMP-OSD assays yielded distinct amplification curves. No increase in OSD signal was observed in duplicate reactions lacking templates (Fig. S1), an especially important finding given the frequent off-target amplification results observed with LAMP lacking OSD probes^39,40^.

**Figure 2:**
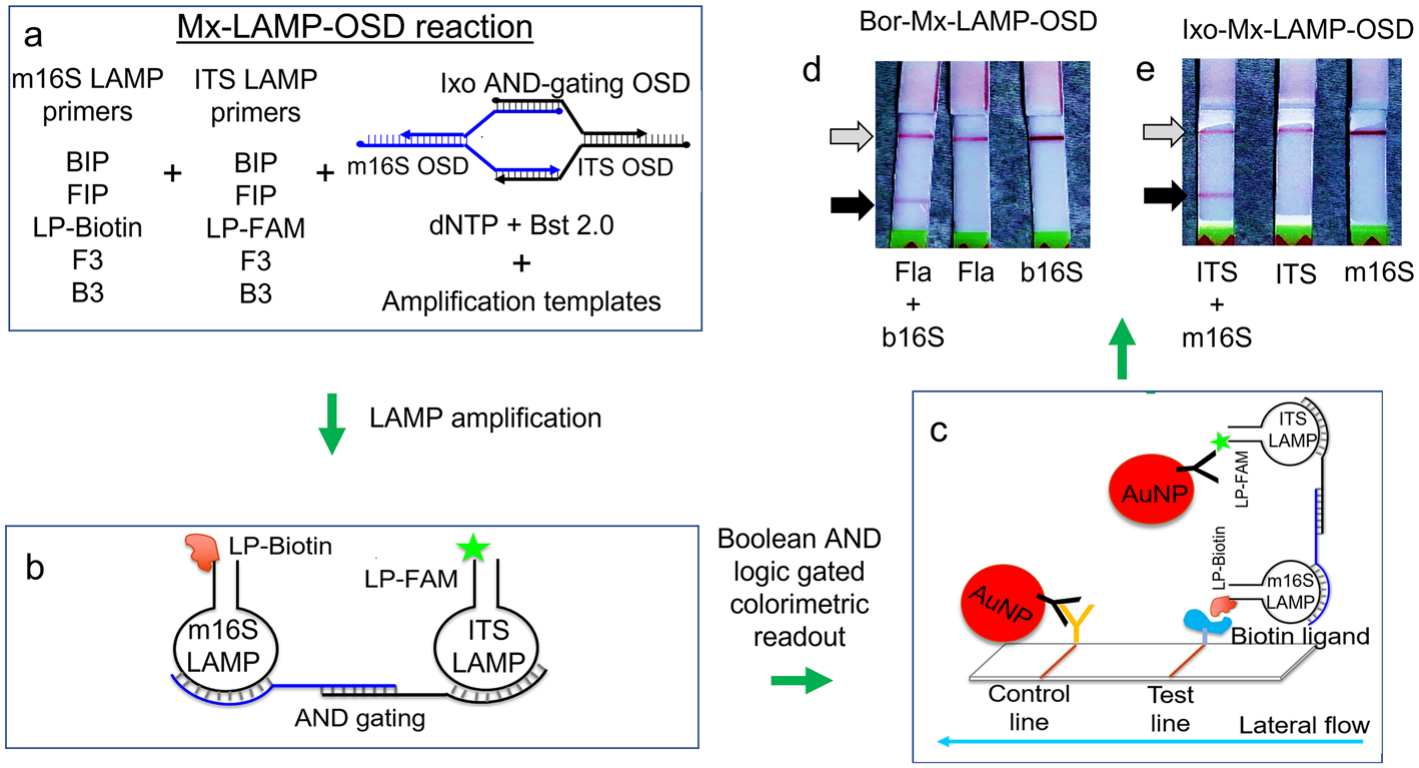
AND-gating of colorimetric readout using Mx-LAMP-OSD assays. (**a**–**c**) Schematic depicting AND gated colorimetric readout using strand displacement probes and multiplex LAMP reactions. (**d** and **e**) Cropped images of lateral flow dipsticks treated with Mx-LAMP-OSD reactions following 90 min amplification of 40,000 copies of indicated synthetic DNA templates for *B. burgdorferi* (**d**) or *I. scapularis* (**e**) are depicted. The gray and black arrows indicate the control and test lines, respectively. Uncropped and multiple exposure versions of the image from which this data was prepared are shown in Fig. S5.

The four sets of LAMP primers were used to create two multiplex LAMP-OSD reactions, one for *I. scapularis* ticks and the other for *B. burgdorferi* spirochaete bacteria. Both the tick-specific and the bacteria-specific amplification reactions were then detected via OSD probes that could bind concurrently to each amplicon, but that only triggered readout when both amplicons were present (a logical ‘AND’ gate; Fig. 1). In greater detail, the *B. burgdorferi* (Bor) and *I. scapularis* (Ixo) LAMP-OSD reactions contained modified loop primers that labeled the Fla amplicons in Bor-Mx-LAMP-OSD or ITS amplicons in Ixo-Mx-LAMP-OSD with fluorescein, and the b16S amplicons in Bor-Mx-LAMP-OSD and m16S amplicons in Ixo-Mx-LAMP-OSD with biotin. Neither single-label amplicon could by itself yield a line on a lateral flow dipstick; only when a fluorescein-labeled LAMP amplicon and a biotin-labeled LAMP amplicon were physically bridged by reactions with their corresponding OSD probes could the resulting dual-labeled assembly be detected (Fig. 2a–c). Co-capturing two amplicons at a single lateral flow test line was a significant advantage over schemes in which two lanes would be required (for example, the separate detection of one amplicon with fluorescein and the other with digoxigenin). While the tick and *Borrelia* multiplex reactions were read out on separate, low-cost, dipsticks with a single test line that captured biotin:fluorescein-labeled analytes, they could potentially be combined in a single, more expensive dipstick that captures biotin:fluorescein and digoxigenin:fluorescein analytes at different lines (such as those available from Milenia Biotec and Abingdon Health, United Kingdom).

**Figure 3:**
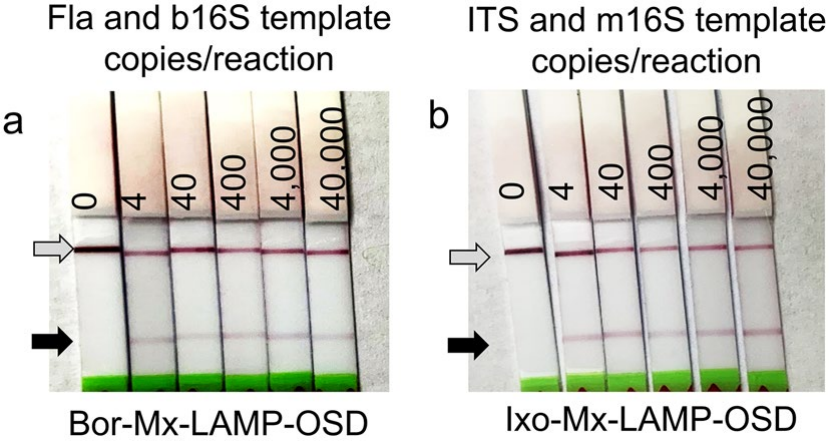
Detection limit of Mx-LAMP-OSD assays. (**a**) Cropped images of lateral flow dipsticks treated with Bor-Mx-LAMP-OSD reactions following 90 min amplification of indicated copies of synthetic DNA templates for *B. burgdorferi* Fla and b16S target sequences. (**b**) Cropped images of lateral flow dipsticks treated with Ixo-Mx-LAMP-OSD reactions following 90 min amplification of indicated copies of synthetic DNA templates for *I. scapularis* ITS and m16S target sequences. The gray and black arrows indicate the control and test lines, respectively. Uncropped and multiple exposure versions of the images from which these data were prepared are shown in Fig. S6.

To verify the AND gating functionality of the Mx-LAMP-OSD assays, duplicate amplification reactions were set up with 40,000 copies of either one or both synthetic DNA templates, representing *B. burgdorferi* and *I. scapularis* samples. After 90 min of LAMP reaction, samples were applied to the lateral flow dipsticks. In both cases, the development of deep red coloration was observed at test lines, but only when both synthetic DNAs were present (Fla and b16S, or ITS and m16S; Fig. 2d and e, respectively). Detection limits were assessed by seeding reactions with 4–40,000 copies of both types of target DNA templates, followed by LAMP amplification and lateral flow colorimetric readout. Again, only assays that included both tick or both bacterial templates developed red coloration at their test zones (Fig. 3). Ultimately, the Mx-LAMP-OSD assays could detect the co-presence of as few as four copies of specific DNA targets without producing non-specific signals.

**Figure 4:**
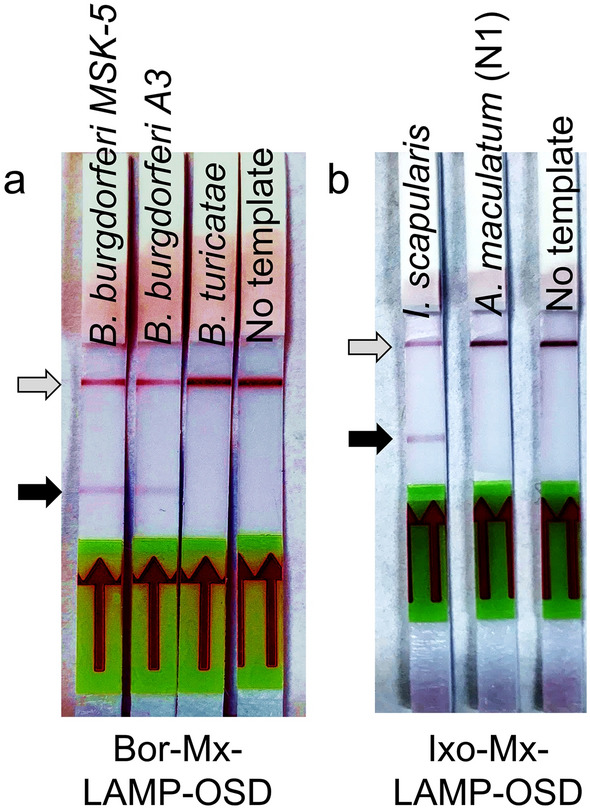
Detection specificity of Mx-LAMP-OSD assays. (**a**) Cropped images of lateral flow dipsticks treated with Bor-Mx-LAMP-OSD reactions following 90 min amplification of indicated genomic DNA templates. (**b**) Cropped images of lateral flow dipsticks treated with *I. scapularis* Mx-LAMP-OSD reactions following 90 min amplification of either macerated *I. scapularis* tick or a non-specific (N1) *A. maculatum* tick. The gray and black arrows indicate the control and test lines, respectively. Uncropped and multiple exposure versions of the images from which these data were prepared are shown in Fig. S7.

The specificity of the assays was further validated by challenging the Bor-Mx-LAMP-OSD reactions with 2 pg of genomic DNA purified either from two different *B. burgdorferi* strains (*Borrelia burgdorferi* A3 and *Borrelia burgdorferi* MSK-5), or from *Borrelia turicatae*, a different *Borrelia* species that can instead cause relapsing fever. Red colorimetric signals were observed for all reactions containing *B. burgdorferi* genomic DNA, but assays that had received the *B. turicatae* sample showed no signal (Fig. 4a). Similarly, the *B. burgdorferi* b16S and Fla LAMP assays yielded bright OSD signals when tested with synthetic DNA templates representative of Fla and 16S rRNA genes of *B. mayonii* and *B. garinii*, suggesting that both these members of the *B. burgdorferi* sensu lato group will be detected by the Bor-Mx-LAMP-OSD assay (Fig. S2). Meanwhile, when these assays were seeded with synthetic *B. miyamotoi* templates only the b16S LAMP assay generated signal while the Fla LAMP reaction failed to activate the OSD reporter and remained dark (Fig. S2). These results indicate that the AND-gated Bor-Mx-LAMP-OSD assay, strictly dependent on co-presence of both16S and Fla amplicons for positive colorimetric outcome (Fig. 2d), would not detect *B. miyamotoi*, a relapsing fever causing *Borrelia* that is also vectored by *I. scapularis* ticks. Similarly, when Ixo-Mx-LAMP-OSD reactions were seeded with 2 µL of crudely macerated material from an *I. scapularis* tick, a deep red colorimetric test signal was observed, while Mx-LAMP assays of a crudely macerated *Amblyomma maculatum* tick specimen yielded negative lateral flow test zones (Fig. 4b). The m16S LAMP-OSD assay also did not produce any signal when tested with synthetic DNA representative of the mitochondrial 16S gene from *I. ricinus, I. persulcatus, and I. pacificus* while the ITS assay produced either no (*I. pacificus*) or a weak (*I. ricinus and I. persulcatus*) OSD signal (Fig. S3). These results indicate that the AND-gated *I. scapularis* Ixo-Mx-LAMP-OSD assay, dependent on reading the simultaneous presence of both ITS and m16S amplicons (Fig. 2e), would not detect *I. ricinus*, *I. persulcatus*, or *I. pacificus*. The LAMP-OSD assays were thus highly specific down to the species level. Most importantly, these data indicate that ticks processed simply by manual maceration can be directly analyzed using Mx-LAMP-OSD assays without any further nucleic acid purification. The elimination of procedures and instruments for nucleic acid separation greatly enhances portability and convenience, and reduces assay cost, enabling both near patient and field surveillance applications.

**Figure 5:**
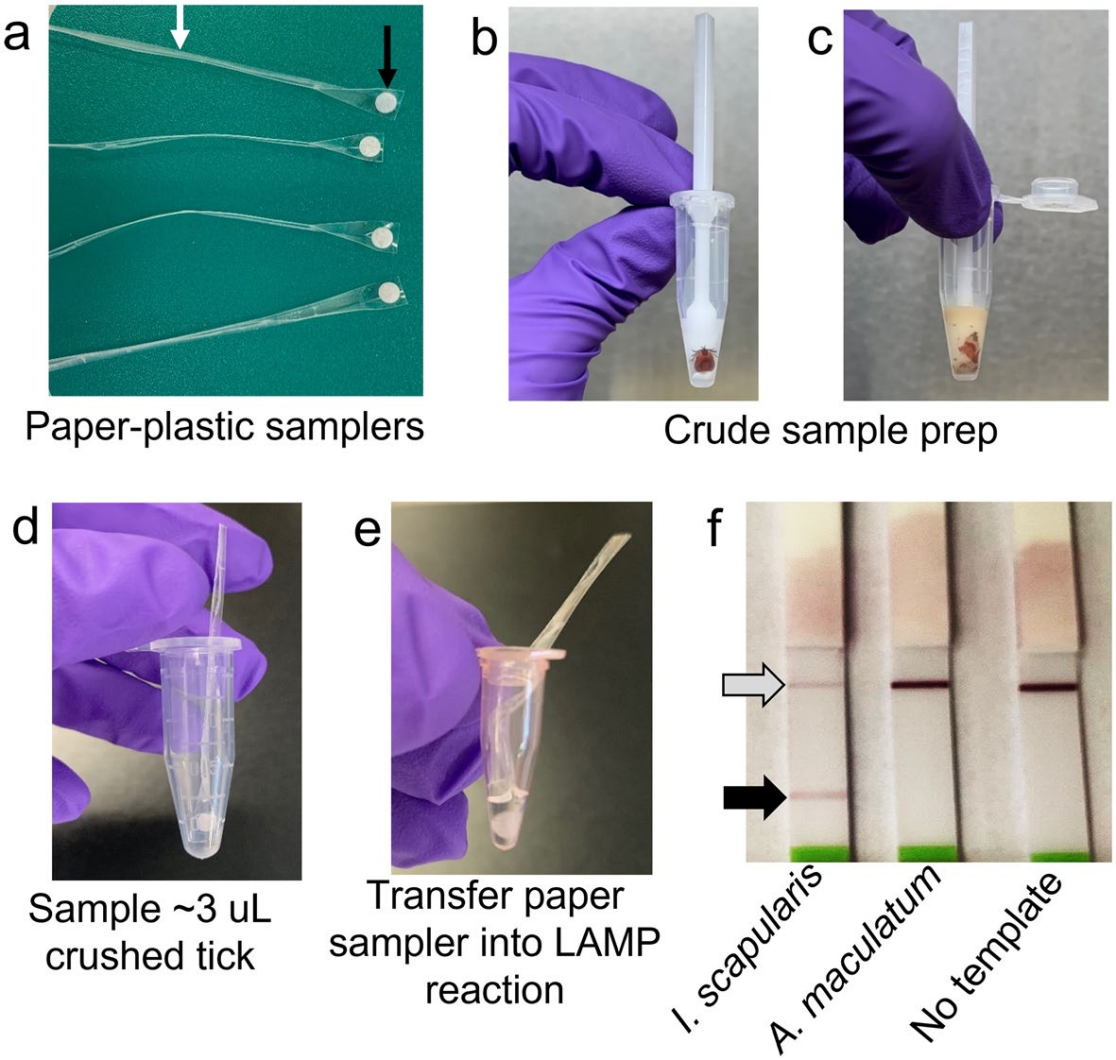
Sample-to-answer tick testing. (**a**) Image of paper-plastic samplers. The black arrow is pointing to the 3 mm glass fiber paper disc. The white arrow is pointing to the handle fabricated from plastic laminating sheet. (**b**) Image of an intact tick placed in a Biomasher II® tube. (**c**) Image of the tick after it was crushed in the Biomasher II® tube. (**d**) Sampling of the crushed tick by immersion of a paper disc sampler. (**e**) Transfer of tick sample into a Mx-LAMP-OSD reaction by dipping the paper disc into the reaction and closing the tube lid over the bent plastic handle to enable incubation for amplification. (**f**) Cropped image of lateral flow dipsticks treated with Ixo-Mx-LAMP-OSD reactions following 90 min of amplification of indicated macerated tick specimens. The gray and black arrows indicate the control and test lines, respectively. The uncropped version of the image from which this data was prepared is shown in Fig. S8.

To further improve accessibility and reduce test cost we sought to demonstrate the feasibility of using a novel methodology to replace sophisticated pipets and barrier tips for transferring tick samples into ready-to-use Mx-LAMP-OSD assay mixes. ‘Disposable samplers’ were assembled by placing a 3 mm glass fiber paper disc at the tip of a thin flexible handle (made by partially folding a strip of plastic laminating sheet; Fig. 5a). To perform a LAMP test with the disposable sampler, a tick was placed in a 1.5 ml Biomasher ® tube (Fig. 5b) and manually macerated for 1–2 min (Fig. 5c), and then a sampler was dipped into the macerated *I. scapularis* or *A. maculatum* tick specimen for 10–15 s. The 3 mm disc soaked up ~ 3 µl of the tick sample (Fig. 5d). The specimen-soaked samplers were then transferred into Ixo-Mx-LAMP-OSD reactions; the assay tubes were capped by bending the flexible handles of the samplers and the 3 mm disc was kept immersed in the reaction during amplification (Fig. 5e). Following 90 min of amplification, the Mx-LAMP-OSD reactions were analyzed on lateral flow dipsticks. Red test lines were observed in assays containing *I. scapularis* specimens, while assays containing *A. maculatum* or no templates were negative (showed white test zones; Fig. 5f). Field-ready paper samplers can thus be used in lieu of pipet and tips for transferring tick specimens into the multiplex reactions, yielding a sample-to-answer platform for testing crudely processed ticks.

### Direct analysis of field-collected and lab-infected ticks using Mx-LAMP-OSD assays and colorimetric readout

**Figure 6:**
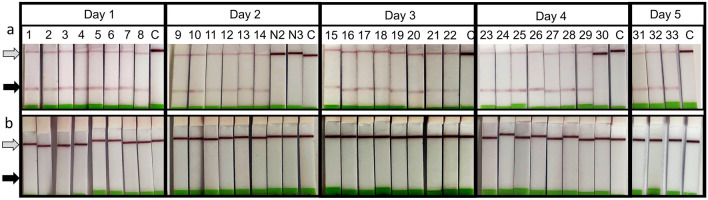
Direct analysis of crudely crushed field collected ticks using *I. scapularis* and *B. burgdorferi* Mx-LAMP-OSD assays. (**a**) Cropped images of lateral flow dipsticks treated with Ixo-Mx-LAMP-OSD tests of *I. scapularis* ticks (numbered 1–33) or *A. maculatum* ticks (N2 and N3). Ixo-Mx-LAMP-OSD test of *A. maculatum* sample N1 is shown in Fig. 4b. Mx-LAMP-OSD assays lacking templates are denoted by ‘C’. (**b**) Cropped images of lateral flow dipsticks treated with Bor-Mx-LAMP-OSD tests of the same tick samples shown in panel a. The gray and black arrows indicate the control and test lines, respectively. Assays performed and imaged on different days are indicated. The uncropped versions of the images from which these data were prepared are shown in Fig. S9.

To validate the sensitivity and specificity of the sample-to-answer Mx-LAMP-OSD assays for field use, direct analyses of a collection of crudely macerated individual ticks was performed. The *I. scapularis* Mx-LAMP assay was assessed with 36 field-collected adult ticks that included 33 *I. scapularis* specimens and 3 *A. maculatum* ticks and with three lab-grown artificially infected and engorged *I. scapularis* nymphs (Table S2). Of the 33 field-collected *I. scapularis* ticks, including some large blood engorged specimens, 32 individuals yielded a positive result as indicated by the appearance of a red colored test line on the lateral flow readout assays (Fig. 6a). Similarly, Ixo-Mx-LAMP-OSD assays of all three lab-grown *I. scapularis* nymphs generated bright red positive signal (Fig. S4). Meanwhile, all 3 *A. maculatum* ticks produced a negative outcome (white test line on the lateral flow readout). None of the field collected ticks showed positive for *B. burgdorferi* when tested with the Bor-Mx-LAMP-OSD test (Fig. 6b). Overall, in these analyses the sample-to-answer *I. scapularis* Mx-LAMP-OSD assay demonstrated a remarkable 100% specificity and 97% sensitivity.

**Figure 7:**
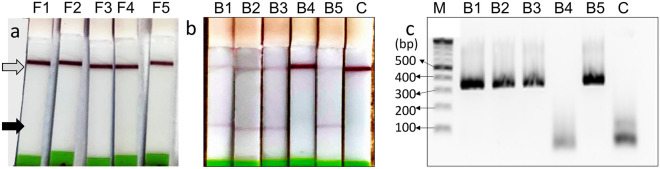
Direct testing of crudely crushed lab-grown ticks with *B. burgdorferi* Mx-LAMP-OSD assays. (**a**) Cropped images of lateral flow dipsticks treated with *B. burgdorferi* Mx-LAMP-OSD tests of uninfected *I. scapularis* ticks (numbered F1–F5). (**b**) Cropped images of lateral flow dipsticks treated with *B. burgdorferi* Mx-LAMP-OSD tests of *I. scapularis* ticks (numbered B1–B5) artificially infected with *B. burgdorferi*. Mx-LAMP-OSD assay lacking templates is denoted by ‘C’. The gray and black arrows indicate the control and test lines, respectively. (**c**) Cropped image depicting agarose gel electrophoretic analysis of nested PCR tests for *B. burgdorferi* ospA gene in the same infected tick samples as used in panel b. M indicates the DNA ladder whose relevant fragment sizes (in base pairs) are indicated with arrows. The uncropped versions of the images from which these data were prepared are shown in Fig. S10.

The *B. burgdorferi*-specific multiplex LAMP-OSD assay was then used to test 10 lab-raised and engorged ticks, five of which were uninfected while the remaining five had been artificially infected with *B. burgdorferi* (Table S2). Assays of uninfected ticks did not result in red colored lateral flow test lines, indicating that these ticks were negative for *B. burgdorferi* (Fig. 7a). Meanwhile, LAMP assays of four of the five infected ticks produced distinct red colored test lines on the lateral flow readout dipsticks indicating that they were positive for *B. burgdorferi* (Fig. 7b). These same individuals also yielded correct 350 bp sized amplicons when assessed by a previously reported nested PCR for *B. burgdorferi* ospA gene (Fig. 7c)^41^. The fifth individual tested negative for *B. burgdorferi* with both multiplex LAMP and ospA nested PCR, likely indicating that its artificial infection by *Borrelia* was not successful and further validating the utility of the LAMP-OSD assay relative to qPCR. Overall, these experiments indicate that the performance of *B. burgdorferi* Mx-LAMP-OSD assay is again highly sensitive and specific, with results comparable to those that might be obtained by a more time-consuming, expensive, and inaccessible PCR-based assay.

## Discussion

Two Mx-LAMP-OSD assays were developed for the identification of *I. scapularis* ticks and *B. burgdorferi* spirochetes in crude macerates relevant to near patient and field surveillance use. The assays incorporated a pair of LAMP primer sets, such that co-presence of the amplicons led to their linkage via sequence specific OSD probes acting as AND gates, and thereby yielded an easy-to-read, visually observable red colored lateral flow test line (positive result). Both diagnostic tests performed robustly with purified DNA templates and demonstrated 97–100% sensitivity and 100% specificity when used for amplification and detection of target sequences directly from crudely macerated ticks, including those engorged with blood, without requiring nucleic acid purification.

These results have significant implications for the detection of LD. In a recent US-wide survey of public health professionals, less than half of the 140 respondents reported conducting regular active tick surveillance in their jurisdictions while only a quarter of the programs were undertaking tickborne pathogen testing^42^. A lack of access to scalable and sustainable easy-to-use molecular diagnostic tools for tickborne pathogen testing was amongst the most cited reasons for this gap. Thus, the availability of a low-cost, easy to use, accurate, and rapid molecular diagnostic tool, such as the Mx-LAMP-OSD assay system, may prove effective in improving public health surveillance as tick geographic distribution continues to expand, with a concomitant rise in the transmission of tickborne diseases.

In addition, this new diagnostic tool may prove useful in improving patient-specific risk assessment and health management, by enabling onsite pathogen testing in ticks or skin rash biopsies removed from bite victims. Individual nymph and adult ticks typically harbor 2 × 10^2^ to 7 × 10^4^ and 6 × 10^2^ to 4.9 × 10^5^ *Borrelia*, respectively^43^. Meanwhile, 10–11,000 spirochaetes can typically be detected per 2 mm skin biopsy by qPCR^44^, a level consistent with the sensitivity of the *B. burgdorferi* Mx-LAMP-OSD assay. Rapid identification of Lyme *Borrelia* in ticks or in patient skin rashes will better allow prophylactic or pre-emptive treatment, and potentially improve patient retention and follow-ups.

The results also have broader and more general implications. The fact that *I. scapularis* and *B. burgdorferi* Mx-LAMP-OSD assays have minimal dependence on centralized laboratory infrastructure and instruments and user required steps opens the way to developing similar assays for other arthropod-borne diseases, such as rocky mountain spotted fever, West Nile disease, tularemia, and Dengue^45^. The processing of multiple analytes by AND-gating OSDs into a single lateral flow colorimetric signal combines simple testing procedures with sophisticated information processing, without the need for complex instrumentation or infrastructure, as we also previously demonstrated for the high surety detection of SARS-CoV-2^39^. While other detection methods (such as labeled primers, molecular beacons, DARQ probes, and even CRISPR-based detection^46–48^) have also been adapted to LAMP, the computational processing power, easy programmability, and robust performance in the presence of biological contaminants continues to argue for LAMP-OSD as perhaps the most versatile and powerful diagnostic system available for near patient and field surveillance use^49–54^.

In conclusion, we have demonstrated the feasibility of facile and accurate sample-to-answer tick testing where crudely crushed ticks are directly added to one pot isothermal nucleic acid amplification reactions and yes/no outcomes are read by simple visual observation of presence or absence of color at assay endpoint. The two prototype assays identify *I. scapularis* ticks or *B. burgdorferi* genospecies by using probes to compute co-presence of two target-derived amplicons thereby suppressing cross-reaction with related organisms, such as *I. ricinus*, *I. persulcatus*, *I. pacificus,* and *B. miyamotoi*. While the current assays have been validated for detecting *B. burgdorferi* genospecies found in North America and ticks prevalent in the mid-Atlantic to eastern United States, these tests could be readily reconfigured for other species. In future work, larger studies will be undertaken to field validate the assays and test protocols with more extensive cohorts of both lab-grown organisms and field collections comprising a wider variety of *Borrelia* and tick species.

## Supplementary Information

Supplementary Information.

## Data Availability

All relevant data have been included within the manuscript and its Supporting Information files.
