# Supplementary material for: Analysis of macerated ticks using Boolean logic gating colorimetric isothermal nucleic acid assays for Lyme Borrelia and Ixodes scapularis ticks
Source: Sci Rep. 2023 Jul 15;13:11439. doi: 10.1038/s41598-023-38452-8 (PMC10349842; doi:10.1038/s41598-023-38452-8)
Supplement: Supplementary file 1 — Supplementary Information. [file 41598_2023_38452_MOESM1_ESM.pdf]

Analysis of macerated ticks using Boolean logic gating colorimetric isothermal nucleic acid assays for Lyme *Borrelia* and *Ixodes scapularis* ticks

Sanchita Bhadra,[1,2] Maria D. Esteve-Gasent,[3] and Andrew D. Ellington[1,2]

Fig. S1

Table S1

Table S2

Fig. S2

Fig. S3

Fig. S4

Fig. S5

Fig. S6

Fig. S7

Fig. S8

Fig. S9

Fig. S10

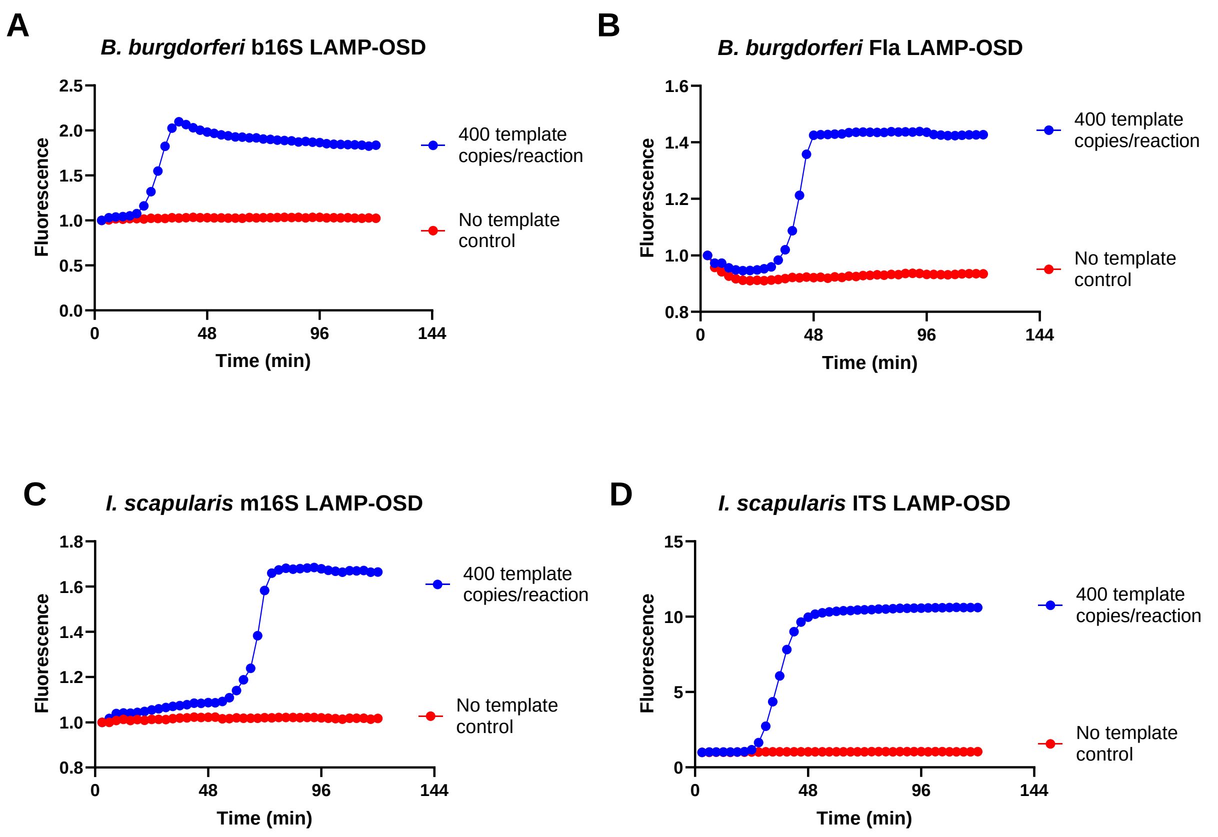

Fig. S1. Real-time amplification kinetics of four individual LAMP-OSD assays for *B. burgdorferi* and *I. scapularis*. Amplification curves generated from real-time measurement of OSD fluorescence in LAMP-OSD assays containing 8 units of Bst 2.0 DNA polymerase and either no specific templates (red traces) or 400 copies/reaction of synthetic DNA templates (blue traces) are depicted. *B. burgdorferi* specific b16S and Fla LAMP assays are depicted in panels A and B, respectively. *I. scapularis*-specific m16S and ITS LAMP assays are shown in panels C and D, respectively.

Table S1. Sequences of primers and probes used in the study.[a]

Name	Sequence	Purpose
Bor.Fla.F3	CTTGAGACCCTGAAAGTGA	Fla assay LAMP primers
Bor.Fla.B3	AATCAGGTAACGGCACAT	
Bor.Fla.FIP	TGCACATGTTATCAAACAAATCTGCTGCTGGTGTGTTAATTTTTGC	
Bor.Fla.BIP	TTGGTTATATTGAGCTTGATCAGCAGATGCAGACAGAGGTTCT	
Bor.Fla.LF	TCTATTAATTTCGTCTGTAAGTTGCTCTATTTCAATTTG	
Bor.Fla.LF.Fam	/56-FAM/TCTATTAATTTCGTCTGTAAGTTGCTCTATTTCAATTTG	
Bor.Fla.OSD.FAM	**TGCATTCCAAG**YTCTTCAGCTGTTYTTACATTYTGCCC/36-FAM/	Fla OSD probes
Bor.Fla.OSD.Q	/5IABkFQ/GGGCARAATGTAARAACAGCTGAAGAR/3InvdT/	
Bor.b16S.F3	TTCCCCGTTTGGGGTCTA	b16S assay LAMP primers
Bor.b16S.B3	CGTGTGTAGCCCAGGACA	
Bor.b16S.FIP	TAACAAGGGTTGCGCTCGTTGCGCATGGTTGTCGTCAGCT	
Bor.b16S.BIP	ACCAGCATGTAATGGTGGGGACACGTCATCCTCACCTTCCT	
Bor.16S.LF	TCAGATAAGACTGCCGGTGATAAGTCGG	
Bor.16S.LF.Bio	/5Biosg/TCAGATAAGACTGCCGGTGATAAGTCGG	
Bor.16S.OSD.FAM	/56-FAM/CACCGTGCTGTGAGGTGTTGG**GTTAAGTCCC**/3InvdT/	b16S OSD probes
Bor.16S.OSD.Q	CCAACACCTCACAGCACGGTG/3IABkFQ/	
Bor.OSD.L1	TGCATTCCAAGYTCTTCAGCTGTTYTTACATTYTGTTCATAAATACTATATTACTAACACT GTCTCGTCTGATCATCGTCAGAGCTG/3InvdT/	Bor-OSD for LFA
Bor.OSD.L2	GGGACTTAACCCAACACCTCACAGCACGGTGTTCATAAATACTATATTACTAACACAGCT CTGACGATGATCAGACGAGACAG/3InvdT/	
Bor.OSD.S1	GTATCTAAGCCGTGTAAAGAGAACATCCTAACTCAATCAATTATATATTATATCARAATGT AARAACAGCTGAAGAR/3InvdT/	
Bor.OSD.S2	GGATGTTCTCTTTACACGGCTTAGATACTAACTCAATCAATTATTTATTATTTCACCGTGC TGTGAGGTGTTGG/3InvdT/	
Ixo.m16S.F3.	AAAATAAGTTCCGTTTTTAGCG	m16S LAMP primers
Ixo.m16S.B3.	AAACCAACCTGGCTTACG	
Ixo.m16S.FIP	TCGAGGTCGCAAACTATTTTATCTATGAAATAAATACTCTAGGGATAACAGC	
Ixo.m16S.BIP	GTTGGATTAGGATTCTTTTTTGGTGACTGAACTCAGATCATGTAGGA	
Ixo.m16S.LR	GAACTATCCAAAATTATTAC	
Ixo.m16S.LR.Bio	/5Biosg/GAACTATCCAAAATTATTAC	
Ixo.m16S.OSD.FAM	**AGAAGTTAAAG**AAAGAAGTTTGTTCAACTTTTAAATCGCC/36-FAM/	m16S OSD probes
Ixo.m16S.OSD.Q	/5IABkFQ/GGCGATTTAAAAGTTGAACAAACTTCTTT/3InvdT/	
Ixo.ITS.F3	CGTGGCGTTGATTTGCATAA	ITS LAMP primers
Ixo.ITS.B3	ACACAAAAGGATGGCGAACT	
Ixo.ITS.FIP	CGCGAAGGACTTAGACTGACGGAGAAAAACGCTTCTGGGAGG	
Ixo.ITS.BIP	TGGAGCCATCCAGTAGGGGAATTGTACACACGCTTTGCATCG	
Ixo.ITS.LR	AAGGCTACGACGCAAAAAACTTT	
Ixo.ITS.LR.FAM	/56-FAM/AAGGCTACGACGCAAAAAACTTT	
Ixo.ITS.OSD.FAM	**GCCGTTGGATTG**CGCGCTTTCTTTTTTGTCGAATCGAAAG/36-FAM/	ITS OSD probes
Ixo.ITS.OSD.Q	/5IABkFQ/CTTTCGATTCGACAAAAAAGAAAGCGCG/3InvdT/	
Ixo.OSD.L1	AGAAGTTAAAGAAAGAAGTTTGTTCAACTTTTAAATCCTTCAATATATCCTATAAATCACA GTATCTAAGCCGTGTAAAGAGAACATCC/3InvdT/	Ixo-OSD for LFA
Ixo.OSD.L2	GCCGTTGGATTGCGCGCTTTCTTTTTTGTCGAATCGACTTCAATATATCCTATAAATCAC AGGATGTTCTCTTTACACGGCTTAGATAC/3InvdT/	
Ixo.OSD.S1	CTGTCTCGTCTGATCATCGTCAGAGCTGTAACTCAATCAATTATATATTATATGATTTAAA AGTTGAACAAACTTCTTT/3InvdT/	
Ixo.OSD.S2	CAGCTCTGACGATGATCAGACGAGACAGTAACTCAATCAATTATTTATTATTTTCGATTC GACAAAAAAGAAAGCGCG/3InvdT/	

[a]FAM: fluorescein; /36-FAM/: 3’-end FAM; /56-FAM/ 5’-end FAM; /invdT/: inverted deoxythymidine; /5Biosg/: 5’-biotin; /5IABkFQ/: 5’ Iowa Black quencher; /3IABkFQ/; Y: C or T; R: A or G.

Table S2. List of field-collected and lab-raised ticks used in the study.

Tick #	Tick species	Development stage (sex)	Origin	Comments
1	*I. scapularis*	Adult (male)	Big Thicket National Preserve, TX	Flat male found on vegetation
2	*I. scapularis*	Adult (male)	Big Thicket National Preserve, TX	Flat male found on vegetation
3	*I. scapularis*	Adult (male)	Big Thicket National Preserve, TX	Flat male found on vegetation
4	*I. scapularis*	Adult (male)	Big Thicket National Preserve, TX	Flat male found on vegetation
5	*I. scapularis*	Adult (male)	Big Thicket National Preserve, TX	Flat male found on vegetation
6	*I. scapularis*	Adult (male)	Big Thicket National Preserve, TX	Flat male found on vegetation
7	*I. scapularis*	Adult (male)	Big Thicket National Preserve, TX	Flat male found on vegetation
8	*I. scapularis*	Adult (male)	Big Thicket National Preserve, TX	Flat male found on vegetation
9	*I. scapularis*	Adult (male)	Big Thicket National Preserve, TX	Flat male found on vegetation
10	*I. scapularis*	Adult (male)	Big Thicket National Preserve, TX	Flat male found on vegetation
11	*I. scapularis*	Adult (male)	Big Thicket National Preserve, TX	Flat male found on vegetation
12	*I. scapularis*	Adult (male)	Big Thicket National Preserve, TX	Flat male found on vegetation
13	*I. scapularis*	Adult (male)	Big Thicket National Preserve, TX	Flat male found on vegetation
14	*I. scapularis*	Adult (male)	Big Thicket National Preserve, TX	Flat male found on vegetation
15	*I. scapularis*	Adult (male)	Big Thicket National Preserve, TX	Flat male found on vegetation
16	*I. scapularis*	Adult (male)	Big Thicket National Preserve, TX	Flat male found on vegetation
17	*I. scapularis*	Adult (male)	Big Thicket National Preserve, TX	Flat male found on vegetation
18	*I. scapularis*	Adult (male)	Big Thicket National Preserve, TX	Flat male found on vegetation
19	*I. scapularis*	Adult (male)	Big Thicket National Preserve, TX	Flat male found on vegetation
20	*I. scapularis*	Adult (male)	Big Thicket National Preserve, TX	Flat male found on vegetation
21	*I. scapularis*	Adult (male)	Big Thicket National Preserve, TX	Flat male found on vegetation
22	*I. scapularis*	Adult (male)	Big Thicket National Preserve, TX	Flat male found on vegetation
23	*I. scapularis*	Adult (male)	Big Thicket National Preserve, TX	Flat male found on vegetation
24	*I. scapularis*	Adult (male)	Big Thicket National Preserve, TX	Flat male found on vegetation
25	*I. scapularis*	Adult (male)	Big Thicket National Preserve, TX	Flat male found on vegetation
26	*I. scapularis*	Adult (male)	Big Thicket National Preserve, TX	Flat male found on vegetation
27	*I. scapularis*	Adult (male)	Big Thicket National Preserve, TX	Flat male found on vegetation
28	*I. scapularis*	Adult (male)	Big Thicket National Preserve, TX	Flat male found on vegetation
29	*I. scapularis*	Adult (female)	Columbus, TX	Engorged female feeding on donkey
30	*I. scapularis*	Adult (female)	Brazos Valley Humane Society	Found feeding on dog at the shelter
31	*I. scapularis*	Adult (female)	Brazos Valley Humane Society	Found feeding on dog at the shelter
32	*I. scapularis*	Adult (female)	Brazos Valley Humane Society	Found feeding on dog at the shelter
33	*I. scapularis*	Adult (female)	Brazos Valley Humane Society	Found feeding on dog at the shelter
N1	*A. maculatum*	Adult (female)	Brazos Valley Humane Society	Found feeding on dog at the shelter
N2	*A. maculatum*	Adult (female)	Brazos Valley Humane Society	Found feeding on dog at the shelter
N3	*A. maculatum*	Adult (female)	Brazos Valley Humane Society	Found feeding on dog at the shelter
F1	*I. scapularis*	Adult (female)	Lab-raised	Pathogen-free and engorged on naïve rabbits
F2	*I. scapularis*	Adult (female)	Lab-raised	Pathogen-free and engorged on naïve rabbits
F3	*I. scapularis*	Adult (female)	Lab-raised	Pathogen-free and engorged on naïve rabbits
F4	*I. scapularis*	Adult (female)	Lab-raised	Pathogen-free and engorged on naïve rabbits
F5	*I. scapularis*	Adult (female)	Lab-raised	Pathogen-free and engorged on naïve rabbits
B1	*I. scapularis*	Nymph	Lab-raised	Artificially infected and engorged on naïve mice
B2	*I. scapularis*	Nymph	Lab-raised	Artificially infected and engorged on naïve mice
B3	*I. scapularis*	Nymph	Lab-raised	Artificially infected and engorged on naïve mice
B4	*I. scapularis*	Nymph	Lab-raised	Artificially infected and engorged on naïve mice
B5	*I. scapularis*	Nymph	Lab-raised	Artificially infected and engorged on naïve mice

Note: Field-collected tick species were identified morphologically.

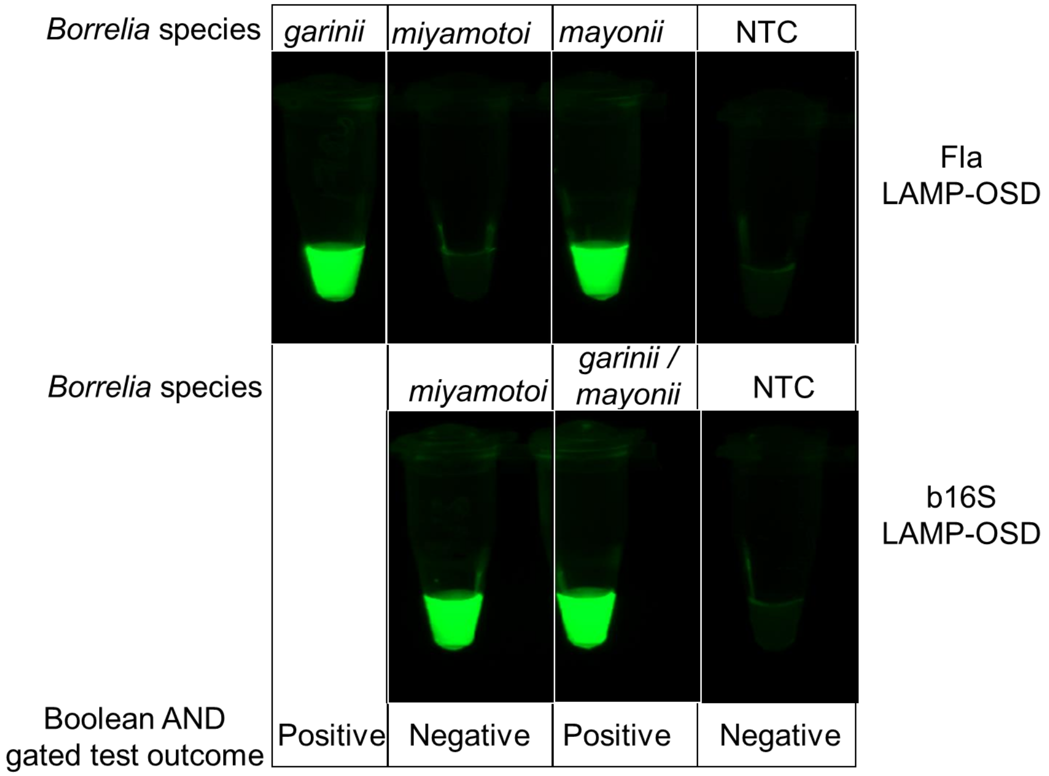

Fig. S2. Detection specificity of *Borrelia burgdorferi* Bor-Mx-LAMP-OSD assay. Images of endpoint OSD fluorescence in b16S or Fla LAMP-OSD assays seeded with 0 (NTC) or 100,000 copies of synthetic DNA templates representative of gene sequences from indicated *Borrelia* species. All assays were incubated for 90 min at 65 °C followed by heat inactivation at 95 °C for 1 min prior to imaging OSD fluorescence using a Gel doc camera.

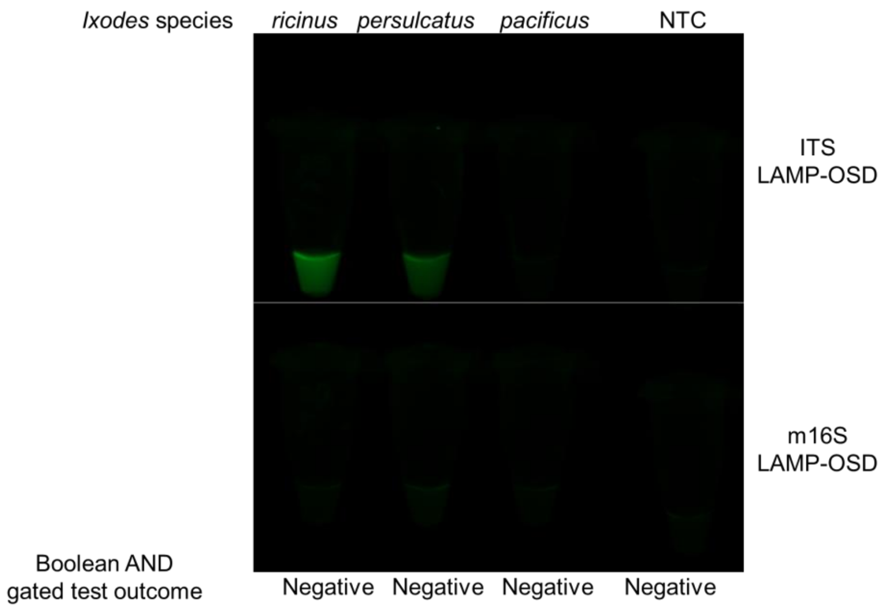

Fig. S3. Detection specificity of *Ixodes scapularis* Ixo-Mx-LAMP-OSD assay. Images of endpoint OSD fluorescence in ITS or m16S LAMP-OSD assays seeded with 0 (NTC) or 100,000 copies of synthetic DNA templates representative of gene sequences from indicated *Ixodes* species. All assays were incubated for 90 min at 65 °C followed by heat inactivation at 95 °C for 1 min prior to imaging OSD fluorescence using a Gel doc camera.

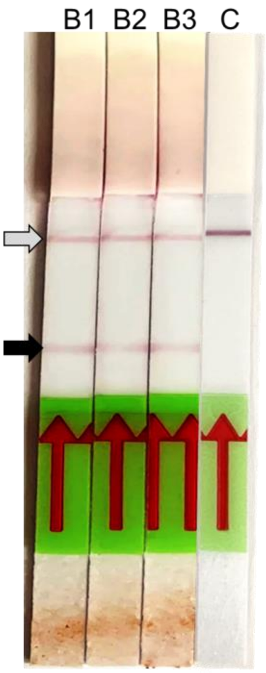

Fig. S4. Direct analysis of crudely crushed lab grown artificially infected and engorged *I. scapularis nymphs* using *I. scapularis* Ixo-Mx-LAMP-OSD assays. Images of lateral flow dipsticks treated with Ixo-Mx-LAMP-OSD tests of *I. scapularis* nymphs (numbered B1 to B3). Ixo-Mx-LAMP-OSD assays lacking templates are denoted by ‘C’. The gray and black arrows indicate the control and test lines, respectively.

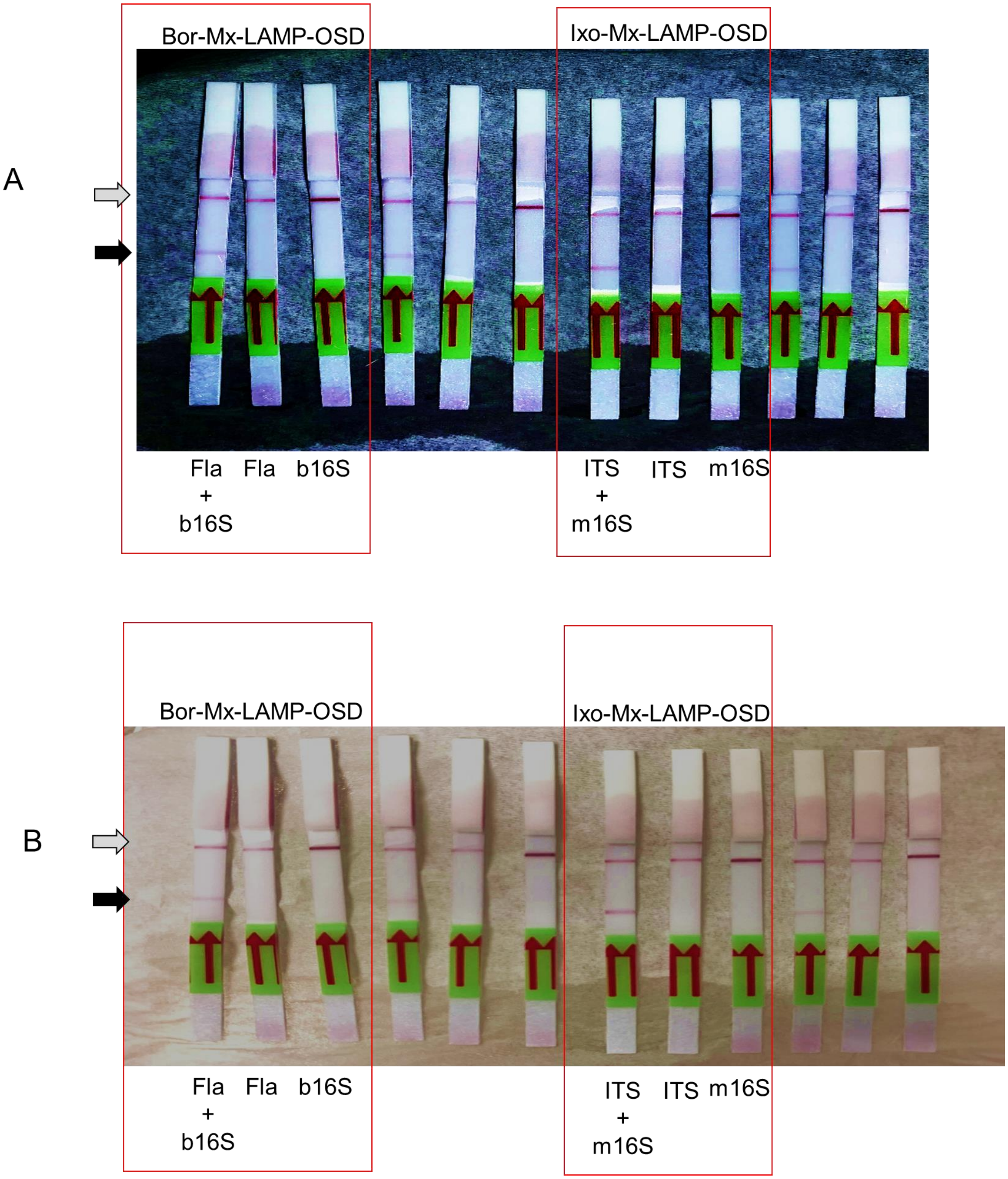

Fig. S5. Uncropped and multiple exposure images for data depicted in Figure 2 of the main manuscript. (A) Uncropped image of lateral flow dipsticks treated with Mx-LAMP-OSD reactions following 90 min amplification of 40,000 copies of indicated synthetic DNA templates for *B. burgdorferi* or *I. scapularis*. Regions of the image within the red boxes were cropped and depicted in Figures 2d and 2e in the main manuscript. The gray and black arrows indicate the control and test lines, respectively. (B) Uncropped image of the same dipsticks as shown in panel A taken with different exposure parameters.

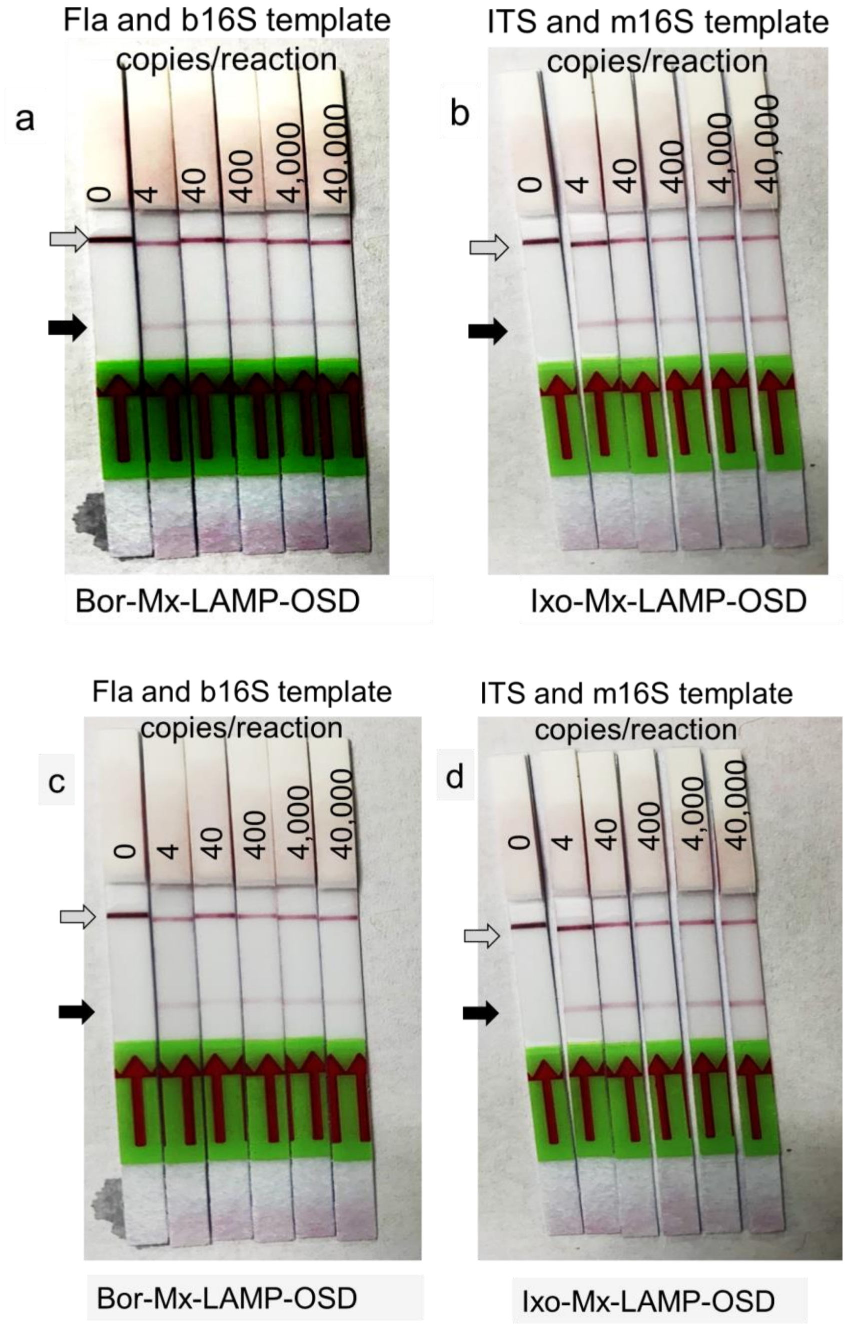

Fig. S6. Uncropped and multiple exposure images for data depicted in Figure 3 of the main manuscript. (A) Uncropped images of lateral flow dipsticks treated with Bor-Mx-LAMP-OSD reactions following 90 min amplification of indicated copies of synthetic DNA templates for *B. burgdorferi* Fla and b16S target sequences (B) Uncropped images of lateral flow dipsticks treated with Ixo-Mx-LAMP-OSD reactions following 90 min amplification of indicated copies of synthetic DNA templates for *I. scapularis* ITS and m16S target sequences. The gray and black arrows indicate the control and test lines, respectively. C and D show uncropped images of the same dipsticks as shown in panel A (C) or panel B (D) taken with different exposure parameters.

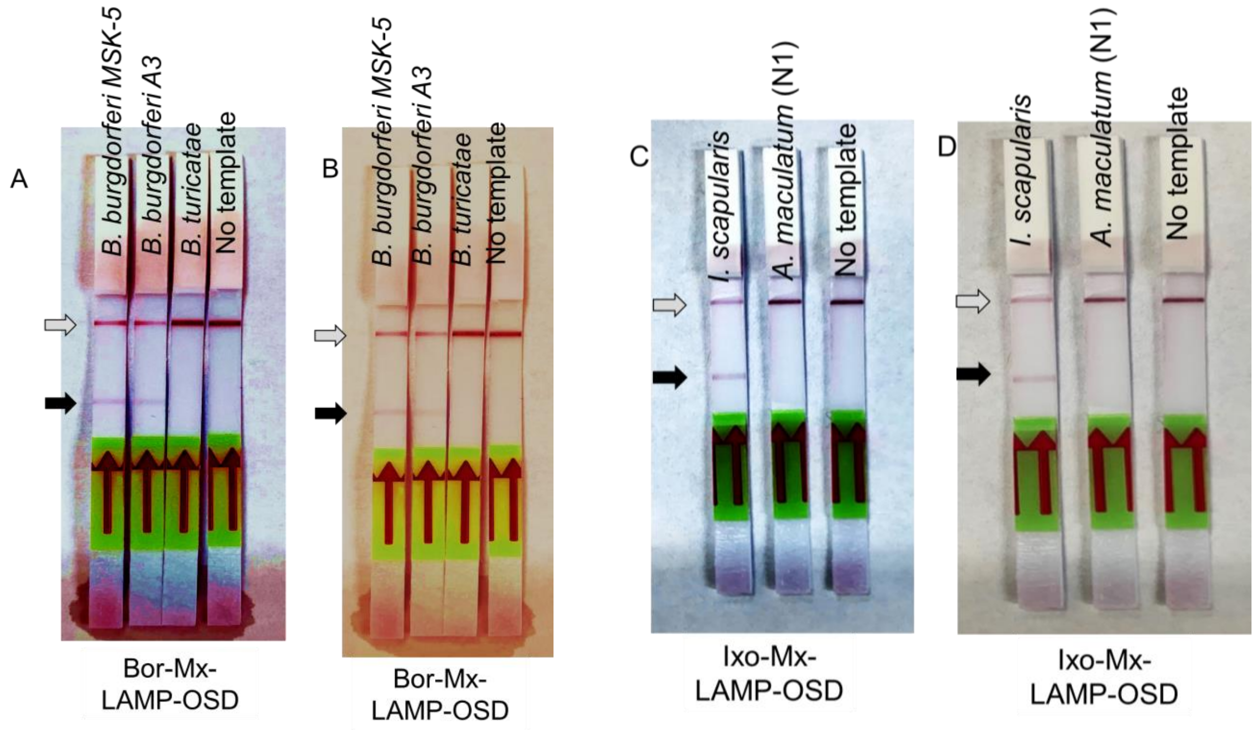

Fig. S7. Uncropped and multiple exposure images for data depicted in Figure 4 of the main manuscript. (A) Uncropped images of lateral flow dipsticks treated with Bor-Mx-LAMP-OSD reactions following 90 min amplification of indicated genomic DNA templates. (C) Uncropped images of lateral flow dipsticks treated with *I. scapularis* Mx-LAMP-OSD reactions following 90 min amplification of either macerated *I. scapularis* tick or a non-specific (N1) *A. maculatum* tick. The gray and black arrows indicate the control and test lines, respectively. B and D show uncropped images of the same dipsticks as shown in panel A (B) or panel C (D) taken with different exposure parameters.

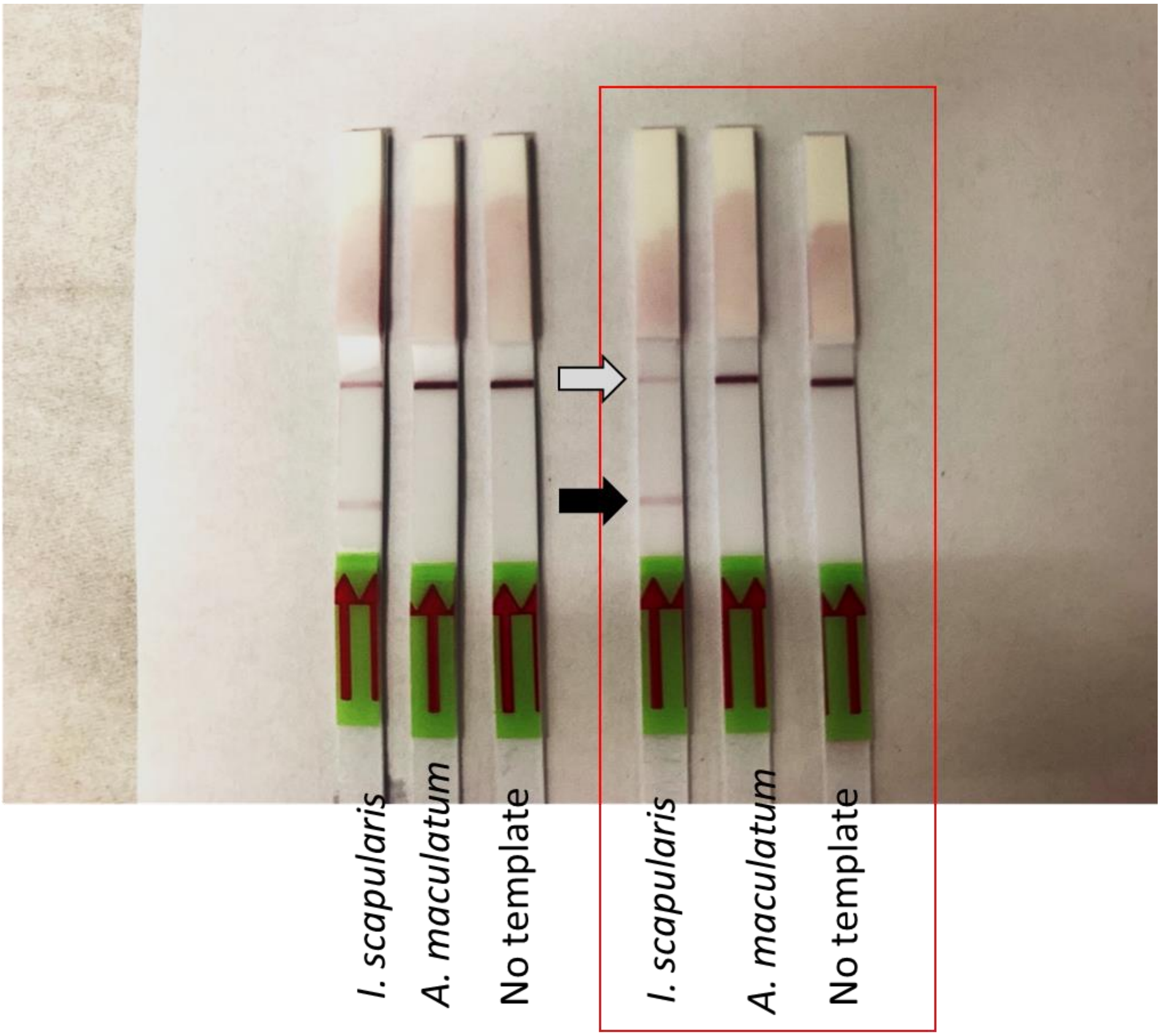

Fig. S8. Uncropped image for data depicted in Figure 5f of the main manuscript. Uncropped image of lateral flow dipsticks treated with Ixo-Mx-LAMP-OSD reactions following 90 min of amplification of indicated macerated tick specimens. The gray and black arrows indicate the control and test lines, respectively. The region of the image within the red box was cropped and depicted in Figure 5f in the main manuscript.

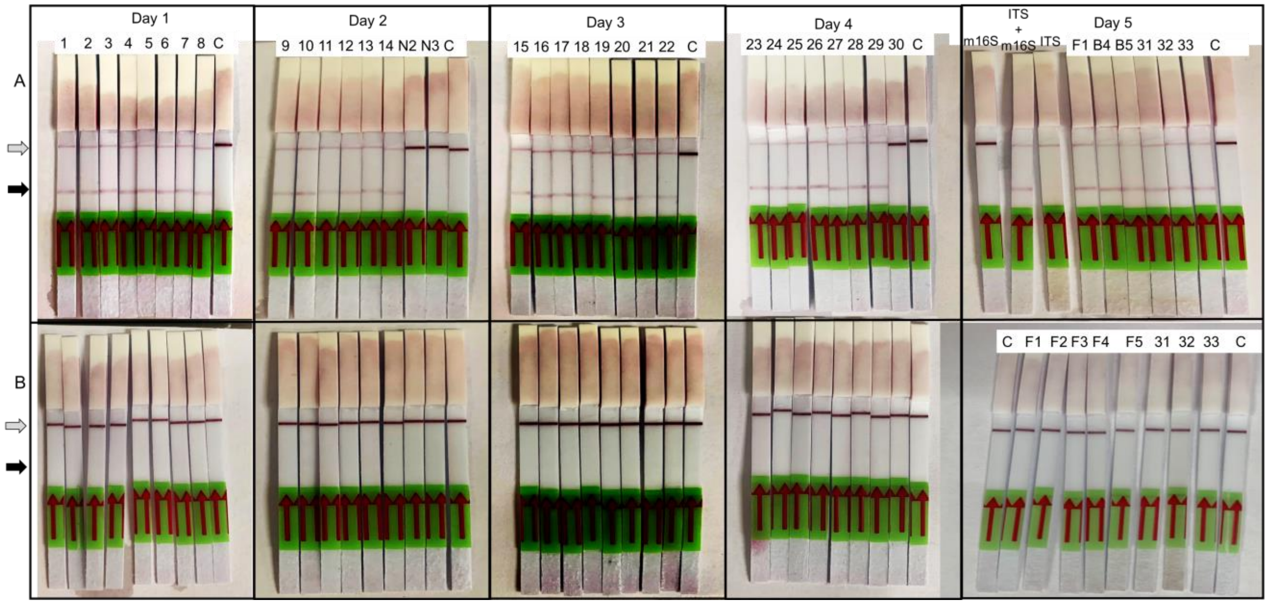

Fig. S9. Uncropped images for data depicted in Figure 6 of the main manuscript. (A) Uncropped images of lateral flow dipsticks treated with Ixo-Mx-LAMP-OSD tests of *I. scapularis* ticks (numbered 1 to 33) or *A. maculatum* ticks (N2 and N3). Ixo-Mx-LAMP-OSD test of *A. maculatum* sample N1 is shown in Fig. 4b of the main manuscript and in Fig. S7. Mx-LAMP-OSD assays lacking templates are denoted by 'C'. Control Ixo-Mx-LAMP-OSD assays seeded with only synthetic templates are indicated in the image of assays performed on day 5. (B) Uncropped images of lateral flow dipsticks treated with Bor-Mx-LAMP-OSD tests of the same tick samples shown in panel A for days 1-4 and of indicated ticks for day 5. The gray and black arrows indicate the control and test lines, respectively. Assays performed and imaged on different days are indicated.

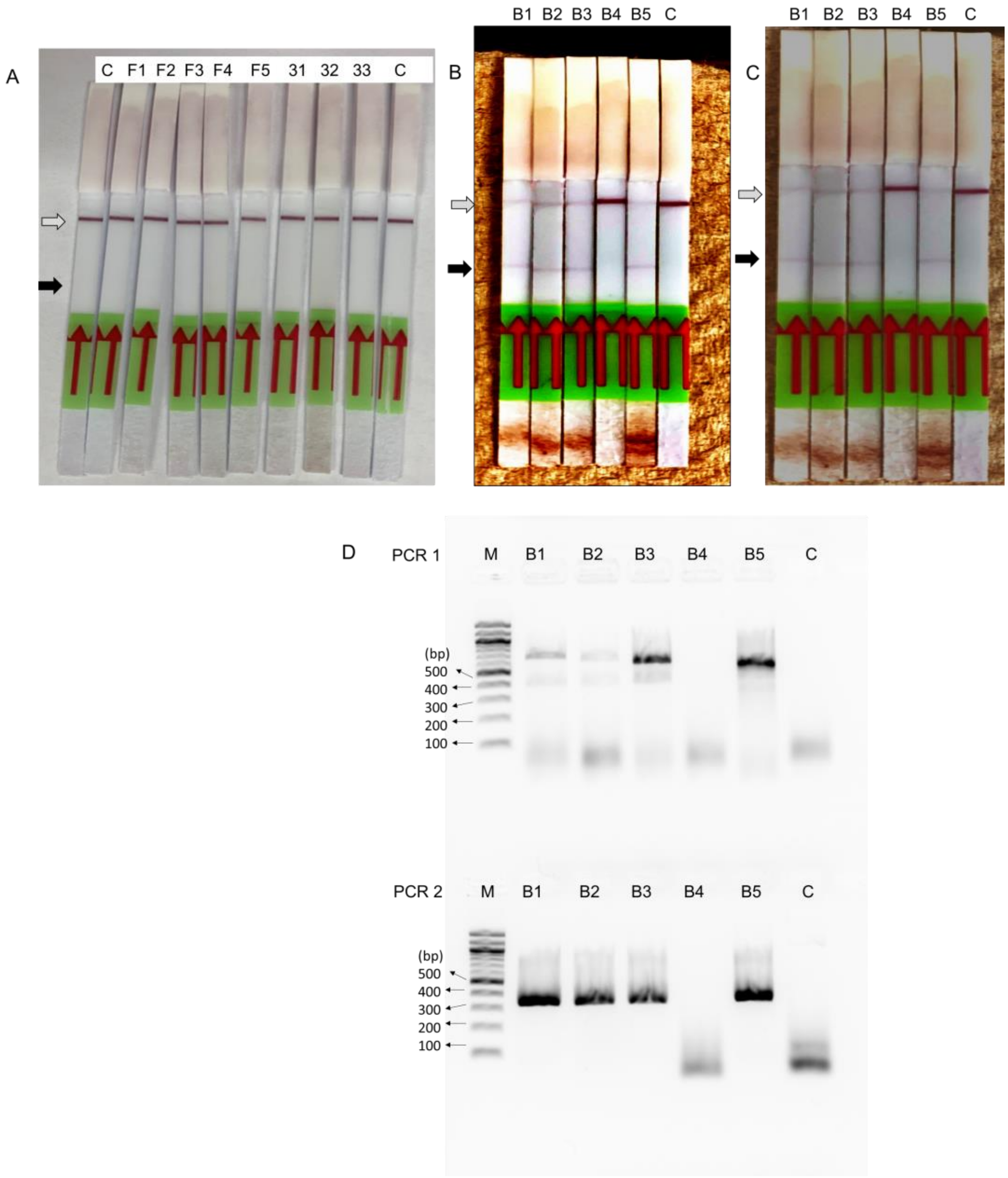

Fig. S10. Uncropped images for data depicted in Figure 7 of the main manuscript. (A) Uncropped image of lateral flow dipsticks treated with *B. burgdorferi* Mx-LAMP-OSD tests of uninfected *I. scapularis* ticks (numbered F1 to F5). (B) Uncropped images of lateral flow dipsticks treated with *B. burgdorferi* Mx-LAMP-OSD tests of *I. scapularis* ticks (numbered B1 to B5) artificially infected with *B. burgdorferi*. (C) Uncropped image of the same dipsticks shown in panel B taken with different exposure parameters. Mx-LAMP-OSD assay lacking templates is denoted by 'C'. The gray and black arrows indicate the control and test lines, respectively. (D) Uncropped image depicting agarose gel electrophoretic analysis of nested PCR tests (top panel: PCR 1; bottom panel: PCR 2) for *B. burgdorferi* ospA gene in the same infected tick samples as used in panel B. M indicates the DNA ladder whose relevant fragment sizes (in base pairs) are indicated with arrows.
